# Extracellular Matrix Formation Enhances the Ability of *Streptococcus pneumoniae* to Cause Invasive Disease

**DOI:** 10.1371/journal.pone.0019844

**Published:** 2011-05-18

**Authors:** Claudia Trappetti, Abiodun D. Ogunniyi, Marco R. Oggioni, James C. Paton

**Affiliations:** 1 Research Centre for Infectious Diseases, School of Molecular and Biomedical Science, The University of Adelaide, Adelaide, South Australia, Australia; 2 Laboratorio di Microbiologia Molecolare e Biotecnologia, Dipartimento di Biologia Molecolare, Università di Siena, Siena, Italy; Instituto Butantan, Brazil

## Abstract

During infection, pneumococci exist mainly in sessile biofilms rather than in planktonic form, except during sepsis. However, relatively little is known about how biofilms contribute to pneumococcal pathogenesis. Here, we carried out a biofilm assay on opaque and transparent variants of a clinical serotype 19F strain WCH159. After 4 days incubation, scanning electron microscopy revealed that opaque biofilm bacteria produced an extracellular matrix, whereas the transparent variant did not. The opaque biofilm-derived bacteria translocated from the nasopharynx to the lungs and brain of mice, and showed 100-fold greater *in vitro* adherence to A549 cells than transparent bacteria. Microarray analysis of planktonic and sessile bacteria from transparent and opaque variants showed differential gene expression in two operons: the *lic* operon, which is involved in choline uptake, and in the two-component system, *ciaRH*. Mutants of these genes did not form an extracellular matrix, could not translocate from the nasopharynx to the lungs or the brain, and adhered poorly to A549 cells. We conclude that only the opaque phenotype is able to form extracellular matrix, and that the *lic* operon and *ciaRH* contribute to this process. We propose that during infection, extracellular matrix formation enhances the ability of pneumococci to cause invasive disease.

## Introduction

Asymptomatic colonization of the upper respiratory tract is the first step in the pathogenesis of infection for most bacteria that cause pneumonia, otitis media (OM) and meningitis [1], [2]. These infections are related to biofilm-like diseases, and more than 60% of bacterial infections are considered to involve microbial growth in biofilms. Indeed, direct detection of biofilms in the nasopharyngeal cavity, in the middle ear mucosa of children with recurrent or chronic OM, and in animal studies, or indirect detection in pneumonia and meningitis, has been demonstrated [3], [4], [5], [6], [7], [8]. A biofilm has been defined as a “microbially derived sessile community characterized by cells that are irreversibly attached to a substratum or interface or to each other, are embedded in a matrix of extracellular polymeric substances that they have produced, and exhibit an altered phenotype with respect to growth rate and gene transcription” [9]. This growth characteristic protects bacteria from many environmental challenges, including antibiotic therapy and host immune defences. Thus, the study of bacteria in biofilm communities will increase our understanding of bacterial pathogenesis and can aid in the development of alternative prophylactic and/or therapeutic strategies.

*Streptococcus pneumoniae* (the pneumococcus) is the predominant pathogen detected in OM cases, followed by *Moraxella catarrhalis* and non-typable *Haemophilus influenzae*, respectively [10]. In addition to being responsible for other local infections like conjunctivitis and sinusitis, the pneumococcus is the predominant cause of community-acquired pneumonia [11]. It is responsible for 1 to 2 million deaths in children under the age of five worldwide, despite the availability of multiple antimicrobials and different vaccine formulations [12], [13]. Pneumococci are also able to undergo spontaneous bi-directional phase variation between two distinct colonial morphologies, described as opaque and transparent. The transparent phenotype exhibits increased *in vitro* adherence relative to opaque variants of the same strain, as well as an enhanced capacity to colonize the nasopharynx of infant rats [14]. On the other hand, the opaque form is associated with massively increased virulence in animal models of systemic disease, and this correlates with increased production of capsular polysaccharide relative to cell wall teichoic acid, as well as pneumococcal surface protein A (PspA), compared with the transparent phenotype [14], [15], [16]. However, to date, the genetic and/or biochemical basis of pneumococcal opacity is yet to be clearly determined. Furthermore, the literature is not clear as to the relevance of the two opacity variants in invasive disease. For instance, the ability to colonize the nasal cavity of infected infant rats was shown to be a predominant characteristic of the transparent phenotype, while no difference was observed in virulence following intraperitoneal injection of either the transparent or opaque phenotype [14]. However, it was later shown that following intraperitoneal infection of adult mice, the opaque phenotype was significantly more virulent than the transparent counterpart [15]. The ability of the two variants to colonize was also tested using a chinchilla model and no difference was observed unless there was a prior challenge with influenza A virus [17]. Finally, it is proposed that the greater recovery of transparent pneumococci from the nasal mucosa of mice is due to their propensity to detach more easily [16].

In addition to colony opacity variants, many other pneumococcal factors have been implicated in colonization and invasive disease [18], [19]. The requirement for capsular polysaccharide (CPS) for colonization was demonstrated in a mouse model by showing that unencapsulated mutants were still able to persist in the nasal cavity, but at a reduced density and duration compared to their encapsulated parent strains [20], [21]. In contrast, unencapsulated strains have been reported to adhere better *in vitro* relative to their encapsulated counterparts [22], [23] Therefore, the relationship between opaque vs transparent phenotype, encapsulation, colonization and biofilm formation are still unclear, and presents an attractive avenue for further study.

While a large body of work has been published on characteristics of biofilm formation in other pathogenic bacteria such as *Pseudomonas aeruginosa* [24], [25] and *H. influenzae* [26], [27], relatively little is known about the characteristics of pneumococcal biofilms and how this impacts on pathogenesis of disease [28], [29], [30], [31]. Given that biofilm formation appears to be the primary mode of pneumococcal growth during the early stages of colonization and invasion, we examined the microscopic architecture of static biofilms formed by a range of clinical isolates starting from pure cultures of opaque and transparent phase variants. The virulence traits of bacteria derived from biofilms formed by transparent and opaque variants were investigated in a mouse intranasal infection model, as was *in vitro* adherence to A549 and Detroit 562 cell lines. The molecular basis for the phenotypic differences was also investigated by microarray analysis of sessile versus planktonic opaque and transparent variants, and the role of candidate biofilm-associated genes in pathogenesis was evaluated in a mouse infection model.

## Results

### Biofilm formation by mixed pneumococcal cultures

**Figure 1:**
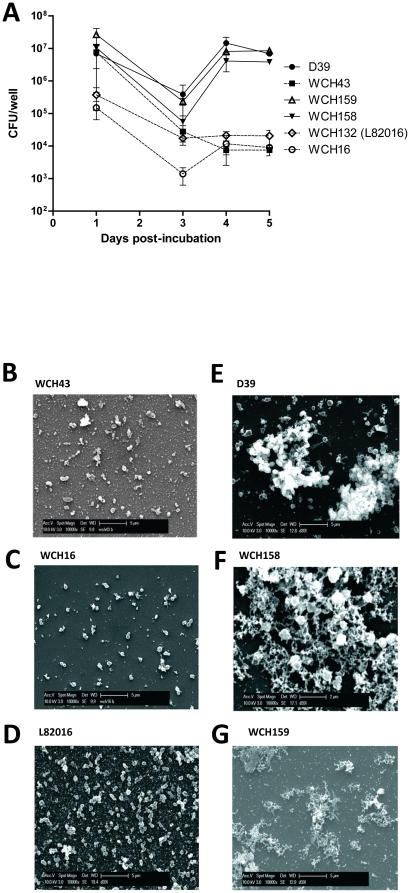
Abilities of various pneumococcal strains to establish biofilm in a static biofilm assay. (**A**), WCH158, WCH159 and D39 were able to form stable biofilm over the incubation period (peaking at day 4), whereas WCH16, WCH43 and WCH132 were impaired in their abilities to form biofilms (*P* = 0.005 and *P* = 0.002 at days 4 and 5, respectively; Students *t*-test; two-tailed). Data for each time point are means ± SEM triplicate samples from a combination of three experiments. (**B**–**G**), SEM analysis showing bacteria attached to the surface in samples of low-biofilm-forming pneumococci, but without matrix (**B**–**D**); and the presence of matrix in samples of high-biofilm-forming pneumococci (**E**–**G**).

A number of *S. pneumoniae* isolates belonging to different serotypes were tested for the ability to form biofilms in a static model in Todd-Hewitt medium supplemented with 0.5% yeast extract (THY). After 24 h incubation, the number of bacteria harvested from sessile cultures was similar for all the strains tested (Fig. 1*A*), ranging between approximately 2×10^4^ to 2×10^6^ CFU/well. At 3 days post-incubation, a decrease in numbers of sessile bacteria was observed for all strains, probably because the bacterial biofilm was in the dispersal stage. However, by day 4, the strains could be split into two groups and an increase in the number of adherent bacteria was observed. This is consistent with a previous study which describes the formation of new biofilm colonies through detachment [9]. The two type 19F strains (WCH158 and WCH159) and the reference strain D39 were able to reach the same density observed at 24 h at days 4 and 5. However, WCH16, WCH43 and WCH132 were still attached to the surfaces as confirmed by enumeration of bacteria, but with 1000-fold lower counts (*P* = 0.005 and *P* = 0.002 at days 4 and 5, respectively). Colony morphology analysis of the cultures on THY-catalase plates showed a higher percentage of transparent variants in the low biofilm-forming strains (WCH16, WCH43 and WCH132), whereas the opposite was the case for the high biofilm forming strains (WCH158, WCH159 and D39). Over time, the transparent variants predominated in low biofilm forming strains, reaching almost 90% at day 4. On the contrary, for high biofilm forming strains, the proportion of transparent variants was always below 50%, and by day 7 the percentage of the opaque variants was almost 90% (Figure S1).

**Figure 2:**
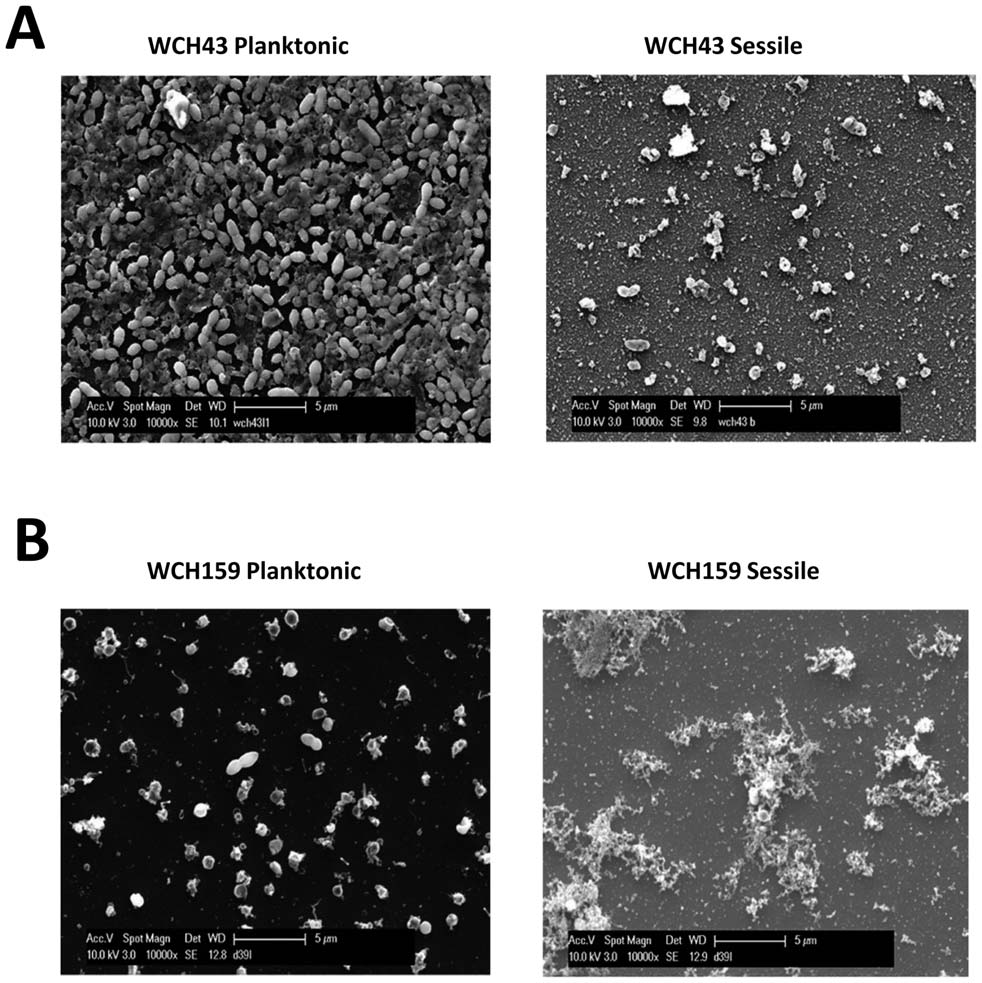
SEM analysis showing: (A), the presence of matrix in the planktonic culture of low biofilm-forming bacteria (WCH43), and (B) the presence of matrix in the sessile culture of high biofilm-forming bacteria (WCH159).

Scanning electron microscopy (SEM) was used to observe the nature of pneumococcal biofilm formation by bacteria grown on coverslips. Sessile and planktonic samples were collected at 4 days post-incubation where the bacterial numbers were higher and when more complex attached microbial communities could be observed. In the high biofilm-forming strains, sessile bacteria were completely embedded in an extracellular matrix (Fig. 1 *B*–*G*), whereas the opposite was the scenario for the low biofilm-forming strains. In the latter, the presence of matrix was observed only in planktonic, but not in sessile bacteria (Fig. 2*A*). The high percentage of transparent bacteria in those samples could lead to easy detachment, because it is likely that the matrix produced by those bacteria was not in sufficient quantity to allow attachment to the substratum. Taken together, these data demonstrate that different pneumococcal strains exhibit distinct biofilm characteristics, and that a link exists between transparent/opaque phenotype and low or high biofilm formation.

### Biofilm formation by opaque and transparent phenotypes and SEM analysis

We observed that strains harvested from static biofilm models showed a clear preference to remain attached to the substratum if the bacterial population was predominantly of the opaque phenotype, whereas the opposite was true if transparent variants were more abundant. Therefore, to investigate the role of the opaque and transparent phenotypes in biofilm formation, pure transparent and opaque colony variants were used and selected as described in Experimental procedures. In the opaque variants, all strains tested were able to form biofilms up to day 7, and no obvious differences were observed over the incubation period. However, the transparent variants of D39, WCH16 and WCH43 were unable to attach to the plastic support, and by day 4, no sessile bacteria were detected (Figure S2). The presence of opaque variants seems to be essential, at least for some serotypes, to permit prolonged adherence onto the plastic substratum. Given the fact that competence has been shown to be involved in the biofilm process [32], CSP-induced competence levels of opacity variants of the six strains used in this study was determined. In all serotypes tested, the transparent variant was more capable of taking up external DNA compared to its opaque counterpart (data not shown). Thus, it appears that the increased ability of opaque variants to form biofilms is not due to increased competence with respect to transparent variants.

**Figure 3:**
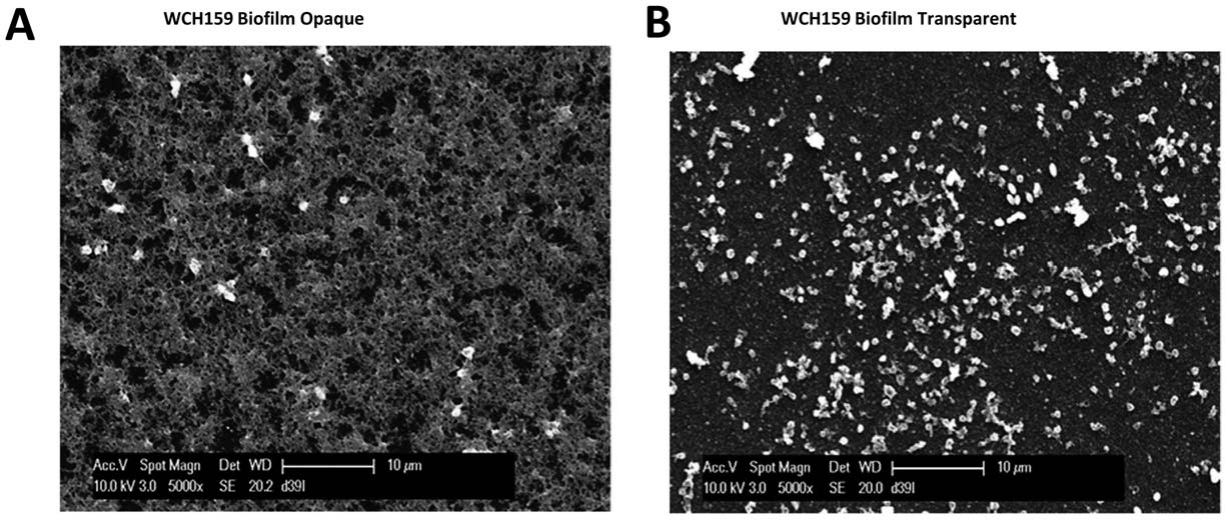
SEM analysis showing: (A), biofilm opaque WCH159 completely embedded in an extracellular matrix and (B), only pure bacteria (no matrix) in the biofilm transparent counterpart.

SEM analysis of strains showed cells embedded completely in an extracellular matrix in the case of sessile samples collected from the opaque variant (Fig. 3*A*), whereas for sessile samples generated by the transparent variants, only matrix-free bacteria were observed (Fig. 3*B*). These analyses clearly show a positive correlation between opaque colony morphology and matrix formation. Therefore, sessile bacteria can be subdivided into two morphological types: those that are able to attach (i.e. form biofilms) but do not produce extracellular matrix, and those form biofilms as well as produce extracellular matrix. We also observed that strain WCH159 was able to form a biofilm from both transparent and opaque variants and showed the most stable phenotypes when comparing different experimental data sets. Therefore, subsequent studies were performed primarily using WCH159.

### Mouse infection model

**Figure 4:**
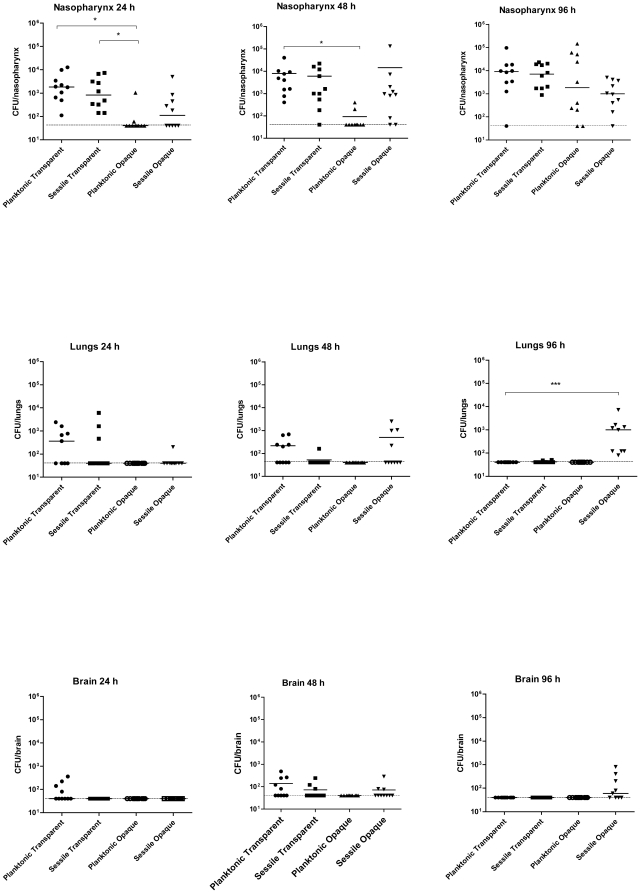
Mouse intranasal (i.n.) infection model. Groups of mice were infected i.n. under anesthesia with the indicated variants of WCH159. At 24 h post-infection, transparent variants adhered to the nasopharynx strongly and at comparable levels, but significantly better than their opaque counterparts (* *P* = 0.03 in both cases). At 48 h, the planktonic and sessile transparent bacteria adhered significantly better to the nasopharynx than the planktonic opaque bacteria (* *P* = 0.048; Students *t*-test; two-tailed). There was a 10-fold increase in the level of nasopharyngeal colonization of mice infected with the transparent phenotype at 4 days post-infection; this difference was not statistically significant between planktonic and sessile forms of the opaque variant. In the lungs and brain, bacteria could only be recovered from mice infected with sessile opaque culture by day 4 (*** *P* = 0.0002; Students *t*-test; two-tailed). Solid horizontal line is median for 9–10 mice per tissue at the indicated time post-infection. Dotted lines represent limit of detection (40 CFU).

A correlation between *in vitro* biofilm formation and *in vivo* infection is already well described [6], [32], [33]. In the current study, we demonstrated the ability of opaque variants to form an extracellular matrix on plastic supports. We then used a mouse intranasal (i.n.) infection model to investigate the significance of matrix formation *in vivo*. For this purpose, CD1 mice were infected i.n. under anesthesia with approximately 5×10^6^ CFU of pneumococci either detached from the surface of microtiter wells or taken from the liquid phase of the culture. At 24, 48 and 96 h post-infection, mice were euthanazed (none of the mice succumbed to infection during this time period) and bacteria from the nasopharynx, lungs, blood and brain of each mouse were enumerated by plating on blood agar, and on THY-catalase plates to verify colony phenotype. As shown in Fig. 4, at 24 h, no difference in bacterial numbers recovered from the nasopharynx was observed between groups of mice infected with sessile or planktonic transparent pneumococci. However, significantly fewer bacteria were recovered from the nasopharynx of mice infected with the planktonic opaque pneumococci with respect to sessile and planktonic transparent pneumococci (*P = *0.03). At all time points, there was no detectable bacteremia, and, with the single exception of mice infected with planktonic transparent pneumococci, no bacteria in the brain at 24 h. The situation observed in the nasopharynx at 48 h was almost the same as at 24 h, except that some bacteria had translocated to the lungs of mice infected with sessile opaque bacteria. At day 4 post-infection, the situation was completely different. Mice infected with sessile opaque pneumococci had bacteria in the lungs, whereas not detectable number of pneumococci could be recovered from mice infected with other phenotypic variants (*P = *0.0002). In the brain, 4 out of 9 mice challenged with sessile opaque bacteria had between 10^2^–10^3^ CFU, although this did not reach statistical significance (Fig. 4). Colony morphology analysis of bacteria recovered from the lungs and brain of mice revealed the presence of a high percentage of opaque variants (Table 1). This result demonstrates that the sessile opaque pneumococci have more ability to disseminate into internal host tissues than their planktonic counterpart. Alternatively, sessile opaque pneumococci most probably have a greater ability to resist phagocytosis than their transparent counterpart. Indeed, this would be expected as it has been shown previously that opaque pneumococci produce more capsule than their transparent counterpart [15].

**Table 1:** Relative percentages of opaque and transparent colonies from bacteria harvested *in vivo*.

Strain	Percentage of opaque phenotype in various niches at the indicated time points:^a^					
	Nasopharynx		Lungs		Brain	
	24 h	96 h	24 h	96 h	24 h	96 h
Wt Opaque	90	85	90	100	-b	96
Wt Transparent	35	25	75	-	75	-
Δ*ciaRH* Opaque	100	100	-	-	-	-
Δ*ciaRH* Transparent	37	48	-	-	-	-
Δ*licD*2 Opaque	85	97	-	-	-	-
Δ*licD*2 Transparent	25	45	-	-	-	-

a Data were obtained by plating bacteria harvested from the indicated niches on THY-catalase plates.

b indicates no bacteria were detected.

### Adherence to A549 and Detroit 562 cell lines by biofilm-forming opaque bacteria

**Figure 5:**
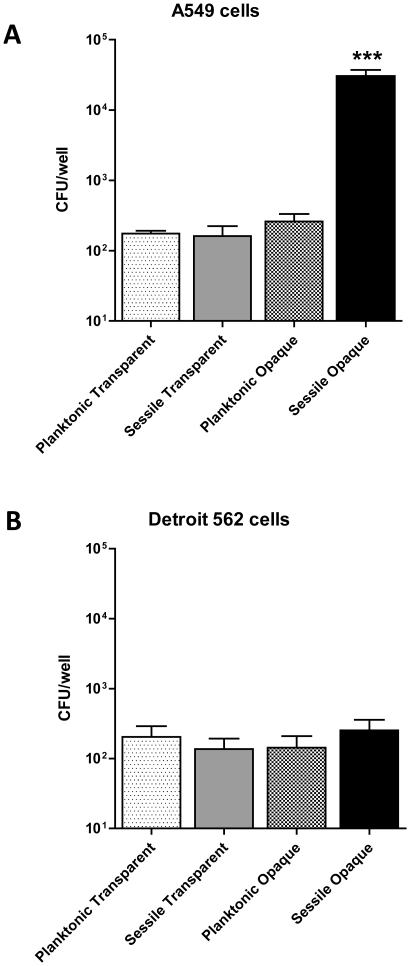
Adherence to A549 and Detroit 562 cells. Planktonic and sessile cultures of transparent and opaque WCH159 grown in a static biofilm model for 4 days, were used in these assays. (**A**), the matrix-producing sessile opaque bacteria were able to adhere to A549 cells at greater than 10^2^-fold more than all the others samples tested (*** *P* = 0.001; Students *t*-test; two-tailed). (**B**), no difference in adherence to Detroit 562 cells between all samples tested. The experiment was performed four times, with similar results. Data are means ± SEM of quadruplicate samples from a combination of three experiments.

The animal infection experiments described above show that the sessile opaque variant of WCH159 could translocate to the lungs and brain of infected mice, whereas planktonic organisms could not. To complement the *in vivo* findings, we investigated whether the matrix produced by sessile opaque bacteria could also promote bacterial adherence to A549 human lung cell monolayers. For this assay, the same planktonic and sessile cultures of transparent and opaque WCH159 grown for the animal challenge experiments were used. As shown in Fig. 5*A*, the matrix-producing sessile opaque bacteria were able to adhere to the A549 cells >10^2^-fold more than all the others cultures tested. To determine whether this was a general phenomenon, we performed the same experiment using a human pharyngeal epithelial cell line (Detroit 562). Interestingly, no difference in adherence was observed between any of the cultures (Fig. 5*B*). It is known that *S. pneumoniae* engages a number of cell surface molecules as targets for diverse adhesins. For example, CbpA binds to polymeric immunoglobulin receptor (pIgR) [34], while phosphoryl choline moieties on teichoic acid recognise the platelet activating factor (PAF) receptor [35]. There are marked differences in the relative expression of these host receptors between cell lines, with Detroit 562 (nasopharyngeal) cells expressing higher levels of pIgR than A549 (type II pneumocyte) cells, while the reverse is true for the PAF receptor [34], [35]. Thus, these data suggests that the matrix-producing sessile opaque bacteria may exhibit a phenomenon specific to some experimental models or cell types.

### Gene expression analysis

To identify the genes responsible for the *in vitro* and *in vivo* phenomena described above, we cross-compared RNA preparations from sessile and/or planktonic cultures from transparent and/or opaque bacteria by microarray analysis. Fully annotated microarray data have been deposited in BµG@Sbase (accession number E-BUGS-117; http://bugs.sgul.ac.uk/E-BUGS-117) and also ArrayExpress (accession number E-BUGS-117). Very few genes were significantly differentially expressed in all combinations analyzed, and this was confirmed by real time RT-PCR (Table 2). In particular, two genes (*ciaR* and *ciaH*) were differentially expressed between sessile and planktonic opaque variants. These genes form part of the same operon, the two component system 5 (TCS05) [36], which had been found to be involved in colonization and adherence in mouse and rat models [37], [38].

**Table 2:** Microarray comparisons of gene expression of *S. pneumoniae* WCH159 transparent (T)/opaque (O) and sessile (S)/planktonic (P) variants.

Gene ID (TIGR4)^a^	Gene ID (G54)^b^	Gene annotation	Fold Change			
			SO/ST	PO/PT	SO/PO	ST/PT
SP_1274	SPG_1168	LicD2 protein	7.6 (100.0)^c^	5.6 (28.6)	(−2.0)	6.2 (1.7)
SP_2108	SPG_2045	Maltose/maltodextrin ABC transporter	(59.0)	7.6 (29.6)	(0.4)	(−49.7)
SP_1580	SPG_1505	Sugar ABC transporter, ATP-binding protein; maltose/maltodextrin transport system ATP-binding	−4.6 (−13.8)	5.3 (9.3)	(−4.1)	(1.0)
SP_0061	SPG_0065	PTS system, IIB component; K02794 PTS system, mannose-specific IIB component	−8.4 (−10.8)	(10.3)	(1.6)	−8.3 (−3.8)
SP_0083	SPG_0084	Two-component system, OmpR family, response regulator	−7.3 (−17.1)	(5.2)	-d	(−1.8)
SP_2148	SPG_2088	Arginine deiminase	−4.3 (−13.0)	(6.3)	(−1.5)	(1.7)
SP_0798	SPG_0728	Two component System 05 Response Regulator (CiaR)	-	-	5.5 (19)	-
SP_0799	SPG_0729	Two component System 05 Histidine Kinase (CiaH)			5.96	
SP_2239	SPG_2188	Serine protease HtrA	-	-	(11.4)	(1.06)

a, b Gene IDs were obtained from the *S. pneumoniae* TIGR4 (serotype 4) and G54 (serotype 19F) genomes as deposited in the Kyoto Encyclopedia of Genes and Genomes (KEGG) database.

c Data in parentheses represent corresponding real time RT-PCR values for the indicated genes.

d No difference in gene expression was observed.

Another comparison that produced an interesting differential gene expression pattern was the sessile transparent versus sessile opaque. In all experiments, the *lic* operon was consistently significantly differentially expressed, being upregulated in the sessile opaque variants. It is already known for other respiratory pathogens such as *H. influenzae* and *N. meningitidis* that the *lic* operon (involved in choline uptake and metabolism) is positively associated with biofilm formation [39], [40], [41]. It has been shown that viable mutants of this operon can only be obtained in the *licD*2 gene, which is required for phosphorylcholine (P-cho) incorporation into lipoteichoic acid [42]. Accordingly, we constructed in-frame mutants of *ciaRH* and *licD*2 to investigate whether the respective mutations can affect adherence and matrix formation.

### Biofilm characteristics of *ciaRH* and *licD*2 mutants

**Figure 6:**
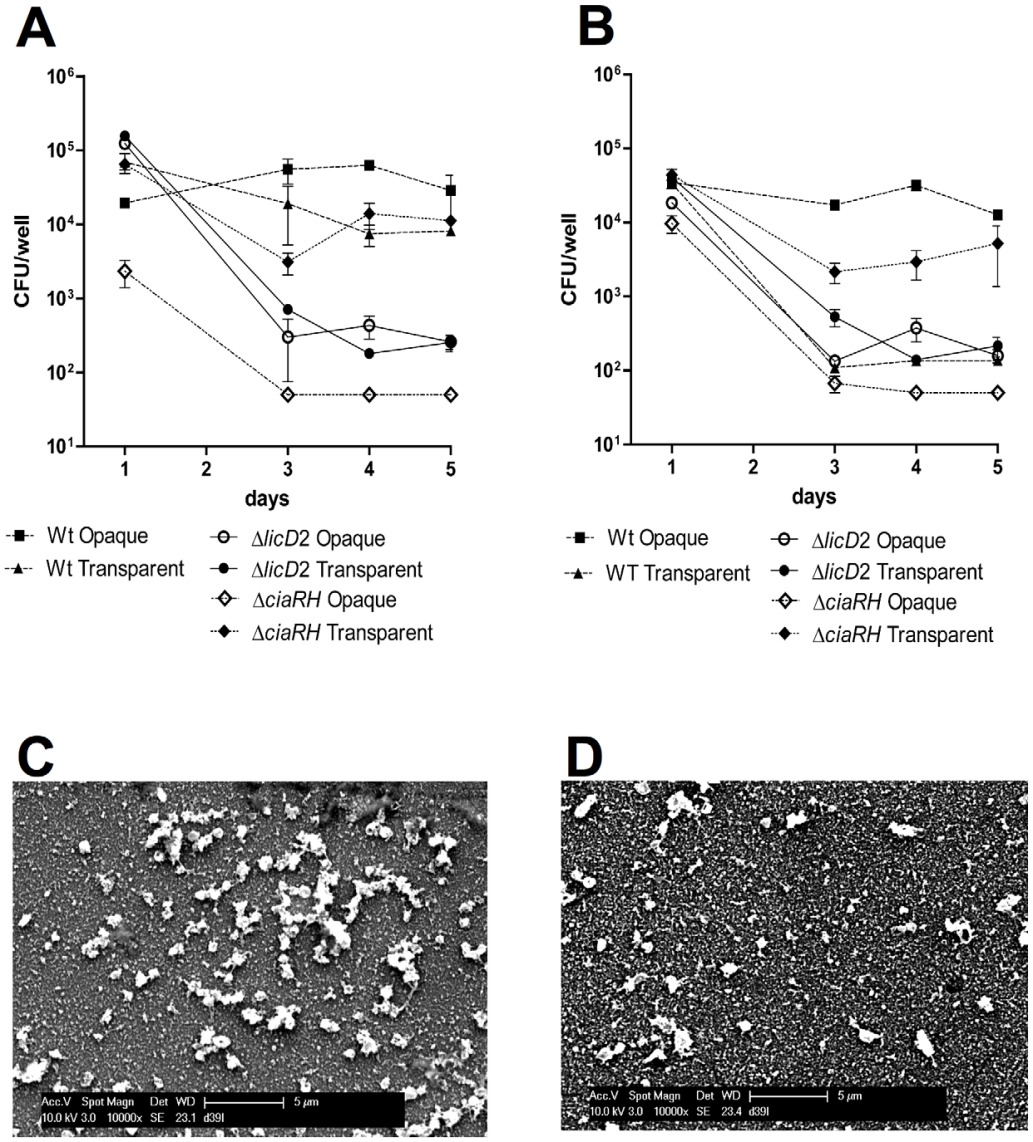
Biofilm and *in vitro* adherence characteristics of Δ*ciaRH* and Δ*licD*2 mutants of WCH159 and D39. (**A**), Static biofilm assay performed with WCH159 derivatives showing that the transparent variant of the Δ*ciaRH* mutant was able to form biofilm to the same level as the wild type, over 5 days, whereas the opaque Δ*ciaRH* mutant could not. However, the Δ*licD*2 mutant showed a significantly low level of biofilm with respect to the wild-type in both phenotypes (* *P* = 0.01 on days 4 and 5; Students *t*-test; two-tailed). Data for each time point are means ± SEM quadruplicate samples. (**B**), Static biofilm assay performed with D39 derivatives; data for each time point are means ± SEM quadruplicate samples. (**C** and **D)**; SEM analysis showing only pure bacteria (no matrix) in the Δ*ciaRH* and Δ*licD*2 mutants, respectively.

The impact of mutating *ciaRH* and *licD*2 was assessed in a static biofilm assay, essentially as described above for wild type bacteria. The numbers of bacteria recovered from pure transparent and opaque biofilms was consistently approximately 100-fold less than what was obtained from mixed culture biofilms. This presumably indicates that the presence of both variants is necessary to achieve high cell density of adherent bacteria. Surprisingly the Δ*ciaRH* mutant showed a different behaviour depending on the phenotype analyzed. As shown in Fig. 6*A*, the transparent variant of the Δ*ciaRH* mutant was able to form biofilm to the same level as the wild type, over 5 days. However, for the opaque Δ*ciaRH* mutant, only 10^2^ bacteria could be recovered after 3–5 days incubation suggesting that a different regulation occurs between the two phenotypic variants. In contrast, the Δ*licD*2 mutant showed a significantly lower level of biofilm with respect to the wild type in variants of both phenotypes (*P<*0.01 on days 4 and 5). Similar results were obtained using Δ*ciaRH* and Δ*licD*2 mutants in D39 background, as shown in Fig. 6*B*. SEM analysis of Δ*ciaRH* and Δ*licD*2 mutants also showed that matrix was absent in both mutants (Fig. 6*C and D*).

### Adherence of *ciaRH* and *licD*2 mutants to A549 cells

**Figure 7:**
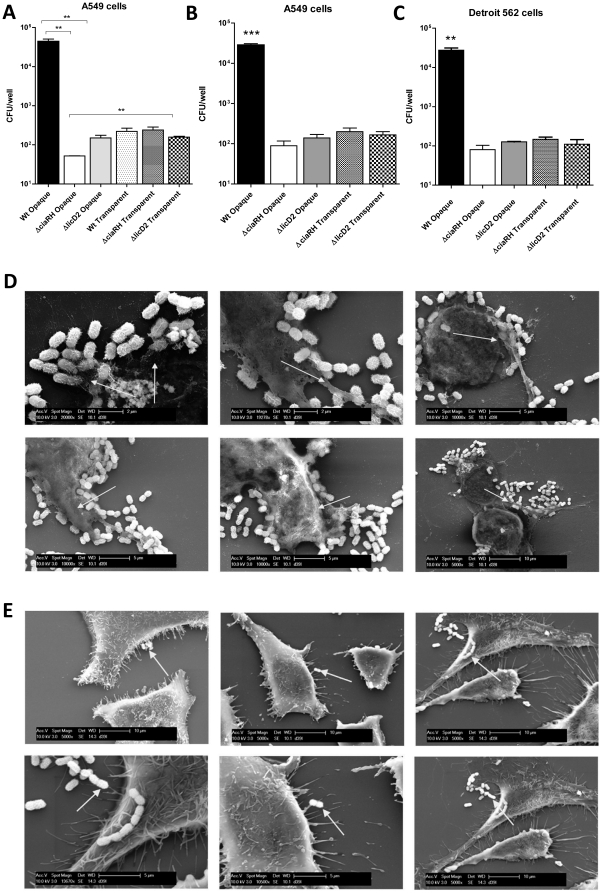
Biofilm and *in vitro* adherence characteristics of Δ*ciaRH* and Δ*licD*2 mutants of WCH159 and D39. (**A**), Experiments performed in WCH159 background; only the matrix-producing opaque wild-type bacteria were able to adhere to A549 cells at greater than 10^2^-fold more than all the others samples tested (*** *P* = 0.003; Students *t*-test; two-tailed). (**B**), Experiments performed in D39 background; only the matrix-producing opaque wild-type bacteria were able to adhere to A549 cells (**B**) and Detroit 562 cells (**C**) at greater than 10^2^-fold more than all the others samples tested (** *P* = 0.003; Students *t*-test; two-tailed). Each adherence assay was performed three times, with similar results. Data are means ± SEM of triplicate samples from a typical experiment. (**D**), SEM showing presence of extracellular matrix and strong attachment of wild-type opaque biofilm bacteria to the surfaces of A549 cells (indicated by arrows). (**E**), No matrix could be seen on A549 cells infected with transparent sessile bacteria. Arrows indicate bacteria only.

Wild type and Δ*ciaRH* or Δ*licD*2 mutants from opaque and transparent biofilm cultures were tested for adherence to A549 cells. For opaque biofilm-derived bacteria, the adherence of the Δ*licD*2 and Δ*ciaRH* mutants was 10^2^-10^3^-fold lower than that of the strongly adherent wild type. In contrast, for transparent phase bacteria, the wild type and Δ*licD*2 and Δ*ciaRH* mutants all exhibited similarly low adherence (Fig. 7*A*). Similar results were obtained using isogenic mutants of D39 on A549 cells (Fig. 7*B*). However, the opaque wild type D39 strain adhered to Detroit 562 cells significantly better than the opaque WCH159 strain (Fig. 7*C*). This could probably be due to the different biofilm architectures, which may be strain-dependent. Alternatively, WCH159 might lack an adhesin that could be present on the surface of D39 cells. SEM analysis of WCH159 adherence to A549 cells showed presence of extracellular matrix and strong attachment of wild type opaque biofilm bacteria (Fig. 7*D*). However, no matrix could be seen on A549 cells infected with transparent sessile bacteria (Fig. 7*E*).

### *In vivo* characteristics of Δ*ciaRH* and Δ*licD*2 mutants

**Figure 8:**
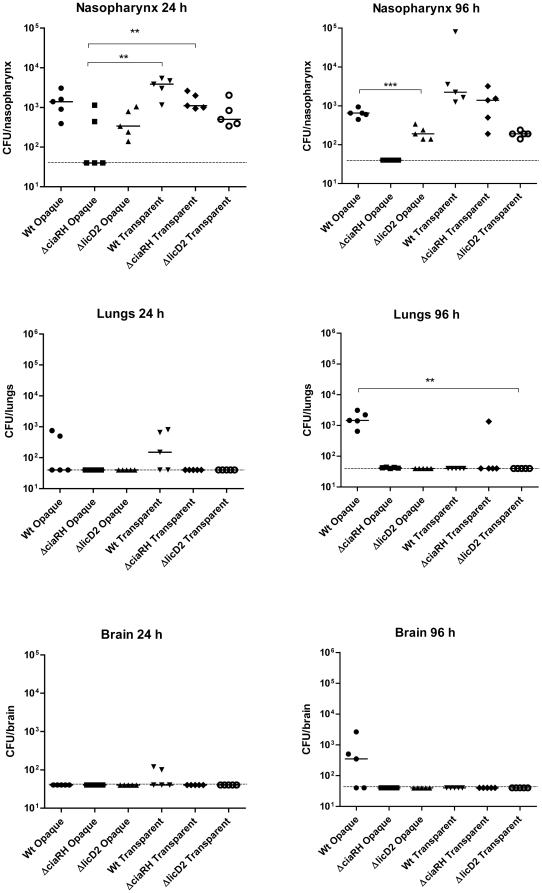
Mouse i.n. infection model showing the effects of *ciaRH* and *licD*2 mutation on their abilities to cause invasive disease. Groups of mice were infected i.n. under anesthesia either with wild-type WCH159 or its otherwise isogenic Δ*ciaRH* or Δ*licD*2 mutant. As shown, with the exception of the opaque Δ*ciaRH* mutant, no significant difference in the colonization of the nasopharynx could be detected at 24 h in all groups tested. However, both phenotypes of the Δ*licD*2 mutant were severely impaired for colonization of the nasopharynx (** *P*<0.001; Students *t*-test; two-tailed). The opaque phenotypes of both mutants were also unable to translocate to the lungs (** *P* = 0.003; Students *t*-test; two-tailed) or the brain (not statistically significant) at 4 days post-infection. Solid horizontal line is median for 5 mice per tissue at the indicated time post-infection. Dotted lines represent limit of detection (40 CFU).

In order to assess the effects of mutating *ciaRH* and *licD*2 on the ability of the biofilm-forming opaque variant to invade the lungs and brain, CD1 mice were challenged with the mutants recovered from static biofilm cultures. The corresponding wild-type biofilm-forming opaque bacteria served as a control. As shown in Fig. 8, there was no significant difference in colonization in nasopharyngeal colonization between all groups, except between transparent Δ*ciaRH* mutant vs opaque Δ*ciaRH* mutant (*P* = 0.02). As observed in the earlier experiment with wild type bacteria, no bacteria were detected in the blood of mice at any time point in this experiment (not shown). As expected, nasopharyngeal colonization by wild type transparent and opaque bacteria was essentially the same level at 24 h and at day 4. The level of colonization of the nasopharynx by the transparent Δ*ciaRH* mutant was also comparable to that of the wild type. However, both phenotypes of the Δ*licD*2 mutant were significantly attenuated for nasopharyngeal colonization with respect to the respective wild type counterpart (*P*<0.001 in both cases). In addition, the capacities of the opaque phenotypes of both mutants to translocate to the lungs and brain were completely abolished at day 4 (*P* = 0.003), although the difference was not statistically significant in the brain.

### Characterization of the extracellular matrix

**Figure 9:**
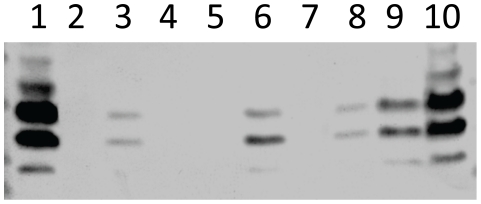
Western blotting of supernatant and pellet (approx 5 × 10^6^ CFU/ml) fractions of samples from a 4-day static biofilm cultures of transparent and opaque WCH159 with TEPC-15 (a mouse anti-phosphorylcholine monoclonal antibody). Lanes: 1, Sessile opaque bacteria; 2, Sessile opaque bacteria, proteinase K-treated for 3 h; 3, Sessile transparent bacteria; 4, Sessile opaque, Δ*licD*2 mutant bacteria; 5, Sessile transparent, Δ*licD*2 mutant bacteria; 6, Sessile opaque bacteria, proteinase K-treated for 1 h; 7, Sessile opaque, Δ*ciaRH* mutant bacteria; 8, Sessile transparent, Δ*ciaRH* mutant bacteria; 9, liquid opaque bacteria; 10, liquid transparent bacteria.

To further characterize the nature of the extracellular matrix produced by the opaque variant, all samples grown in the static biofilm model were electrophoresed on SDS-PAGE and immunoblotted with TEPC-15 (a mouse anti- P-cho monoclonal antibody). As shown in Fig. 9, we detected more choline associated with teichoic acid (TA) in opaque sessile samples, and it was completely absent in the two mutants. This result is in accordance with *licD*2 upregulation, and indicates that TA might be part of the extracellular matrix. Furthermore, the differential centrifugation showed that the supernatant fraction is composed almost exclusively of the matrix, because it was devoid of bacteria upon subsequent plating on blood agar (Figure S3). As shown, addition of proteinase K treatment to the samples resulted in partial loss of the band intensity. DNase and proteinase K treatment of samples from WCH159 and D39 also resulted in partial degradation of the matrix (result not shown). Congo red staining of cultures also showed colour change from red to pink-orange for the sessile opaque strains (not shown). These results suggest that the matrix formed by these strains is similar to that described by others [6], [43], [44].

### Capsule ELISA

To determine whether some of the differences observed between the strains are related to changes in capsule regulation or amount, we measured the amount of capsule in the variants of WCH159 and their isogenic mutant derivatives. Our measurements show that total amount of capsule in all the strains are essentially similar (approx. 10 ng total capsule/ 10^9^ cells).

## Discussion

Despite the many studies on biofilm formation in *S. pneumoniae*, it is still not completely clear why some serotypes are more likely to form a biofilm *in vitro* or *in vivo*, or whether a correlation exists between biofilm formation *in vitro* and ability to cause invasive disease [28], [29]. Some lines of evidence have demonstrated that *S. pneumoniae* can form biofilms *in vivo* and are able to form a sticky matrix on the mucosal surface, although not all pneumococcal serotypes are able to invade the host and cause disease [6], [45]. By starting with pure cultures of opaque and transparent phenotypes, we have demonstrated that the ability to form matrix is an exclusive feature of the opaque variant and that this phenomenon shows strain-specific variations. Static biofilm assays carried out on transparent and opaque variants of pneumococci allowed us to separate strains into low and high biofilm formers, with a high proportion of transparent variants in the low biofilm-forming group. The transparent variants of some strains failed to form a biofilm after 2 days incubation, whereas the opaque variants of all the strains were able to remain attached to the plastic surfaces up to 5 days. Our results suggest that the opaque phenotype seems to be indispensable to maintaining bacterial aggregation and attachment to the surface. The explanation for this phenomenon was found when sessile and planktonic bacteria of transparent or opaque phenotypes were analyzed by SEM. Surprisingly, we found the presence of abundant extracellular matrices only in the opaque, sessile samples, whereas the transparent variants were unable to build an extracellular matrix in any of serotypes tested.

Biofilm has been reported to be involved in the pathogenesis of other bacterial diseases. For example, in *P. aeruginosa*, a Gram-negative pathogen responsible for a wide range of infections including lung infection in cystic fibrosis patients, biofilm and consequent production of matrix is the major virulence factor [46]. In oral streptococci, the extracellular matrix is directly involved in adherence to dental surfaces during the development of dental caries [47], [48]. Furthermore, *Staphylococcus aureus*, which is responsible for frequent catheter- or prosthesis- derived infections, is extremely difficult to treat due to the matrix which it is able to build [49], [50]. Finally, *H. influenzae*, which also causes pneumonia, meningitis and OM, shares many aspects of biofilm formation and extracellular matrix production as pneumococci [51], [52].

To investigate how the bacteria embedded in an extracellular matrix are involved in the pathogenesis of pneumococcal disease, we infected mice with bacteria collected from sessile and planktonic morphs of transparent and opaque variants [32]. Only groups of mice infected with opaque sessile pneumococci showed infection of the lungs (and brain) at 4 days post-infection, and these findings were supported by A549 adherence assay. In fact, A549 cells infected with opaque and transparent planktonic bacteria showed no difference in adherence. This is in agreement with another study, which showed that planktonic opaque and transparent pneumococcal variants adhered to a similar degree to non-activated epithelial and endothelial cells [53]. In contrast, sessile opaque bacteria adhered 1000-fold more to the A549 cells than all the other samples, suggesting a role for the matrix in the process. Our findings showing stronger binding of opaque biofilm-derived bacteria to A549 cells (but not to Detroit 562 cells) is as expected, given that the biofilm-associated matrix contains a large amount of TA, thereby promoting strong interaction with the PAF receptor expressed by the former cell type [34], [35]. Improved adherence to lung cells is consistent with progression to invasive disease. Likewise, the lack of a statistically significant difference in longer-term nasopharyngeal colonization is consistent with the Detroit 562 adherence data.

In our attempt to understand the molecular basis for the observed phenomenon, we used microarray analysis to investigate the gene expression profiles of planktonic and sessile bacteria from transparent and opaque variants. The analysis showed regulation principally in two operons: the *lic* operon, which is involved in choline uptake, and the *ciaRH* operon, the first two-component system to be characterized in *S. pneumoniae* [36]. The *lic* operon plays a key role in the survival of pneumococci, as a deletion in this operon is lethal [54]. Choline is taken up from medium and transported inside of the cell by Lic1, converted to P-cho, and then incorporated into the TA chains by *licD*2 [42]. P-cho occurs as cell-wall-associated and lipotheichoic acid, and is a surface component of many mucosal pathogens, such as *Neisseria* and *H. influenzae*. These bacteria reside in the same biological niche and share an identical gene set for choline uptake and incorporation (*licA*–*D*). In these bacteria, *lic* expression correlates positively with biofilm maturation [39], [41], [55], [56]. In this study, the operon was highly up-regulated in bacteria collected from opaque samples consisting of more complex attached microbial communities. Upregulation of the operon was further confirmed by RT-PCR analysis of *licA* and *tacF* (Table S1), which showed upregulation of both genes in the sessile opaque bacteria (as seen with the expression of *licD*2). These results are consistent with findings in other mucosal pathogens such as *Neisseria* and *H. influenzae*. We also determined relative expression of choline binding proteins (CBPs) *cbpA*, *lytA*, *pcpA*, and *pspA* between RNA samples from sessile opaque and sessile transparent bacteria. Interestingly, the expression of all these genes was much higher in RNA samples from the sessile transparent bacteria (Table S1).

To further investigate the role of the *lic* operon in pneumococcal biofilm formation and pathogenesis, we constructed a Δ*licD*2 mutant, this being the only gene in the *lic* operon that is not lethal, and results in a decrease, but not total abolition of choline uptake [42]. The mutant was unable to form a stable biofilm in both transparent and opaque variants; both variants were also impaired in nasopharyngeal colonization and ability to translocate to the lungs or brain at all time points. In addition, the level of adherence of the opaque Δ*licD*2 mutants to A549 cells was massively diminished relative to the opaque wild type, and comparable to that of the transparent wild type variant. Interestingly, it was reported that the *lic* operon was upregulated in CSF cultures and in pneumococci adherent to epithelial cells, and there was an enhanced expression of the *ciaR/H* genes during endothelial cell contact [57].

The *in vitro* and *in vivo* effects of *licD*2 mutation observed here correlate with those seen with *licD*2 mutants of non-typeable *H. influenzae* [4], [39]. Decrease in ability to incorporate P-cho into the TA chains correlates with inhibition of matrix formation, capacity of cause invasive disease as well as adhere to lung cells. Furthermore, the *ciaRH* operon was more highly expressed in opaque sessile bacteria compared to their planktonic counterpart. The *ciaRH* two-component system has previously been found to be the only two-component system in *S. pneumoniae* that is positively involved in colonization and related to biofilm [36], [38]. In our static biofilm model, the opaque *ciaRH* mutant was unable to maintain a stable biofilm beyond two days, whereas the transparent counterpart showed the same level of biofilm as the wild type, up to five days. In the mouse model, colonization of the nasopharynx by the transparent *ciaRH* mutant was comparable to wild type levels, while the opaque *ciaRH* mutant was unable to colonize the nasal tissue beyond 24 h. Essentially similar results were obtained in the A549 adherence assay. Since it is already known that *ciaRH* positively regulates the expression of *htrA* [37], we investigated the expression of this gene by real time RT-PCR. Our data shows that there was a concomitant increase in *htrA* expression in opaque sessile sample, where the expression level of *ciaRH* was also upregulated. However, these findings contrast with a recent report of the phenotypic effect of *ciaRH* mutation on expression of *htrA* in transparent and opaque variants [58]. In that study, transcriptional analysis of *htrA* levels in opacity variants demonstrated that transparent strains produce more *htrA* transcript than the opaque variant during late-exponential phase. This discrepancy could be due to the fact that in our study, analysis was carried out on bacteria collected from a 4-day old biofilm assay rather than from bacteria grown in rich broth to late exponential phase. Consistent with this, we found by RT-PCR analysis that *htrA* expression in late exponential phase cultures of transparent bacteria was higher than in opaque bacteria (Table S1).

In *S. pneumoniae*, *ciaRH* has been shown to positively regulate expression of the *lic* operon [37] which is responsible for P-cho uptake and incorporation onto TA. Choline has been shown to be present in pneumococcal biofilm matrix [43]. Thus, it may be expected that the molecular mechanism behind the opaque associated-matrix phenomena is related to increased ability of opaque variants to process choline through the *lic* system. Choline is needed for many physiological functions in the human body such as building of cell membranes and movement of nutrients between cells, but most importantly, plays a key role in the brain. It is a precursor for acetylcholine and membrane phosphatidylcholine, and is obtained principally by dietary intake and transported across the blood-brain barrier through facilited diffusions [59], [60]. Meningitis-associated and biofilm-forming pathogens such as *N. meningitidis*, *H. influenzae* and *S. pneumoniae* are able to take up choline and then process and display it on the surface. The importance of surface display of choline residues on the surface of *H. influenzae* is well characterized [61]. By using novel fluorescent reporter systems, membrane localization of choline-utilization (Lic) proteins in *S. pneumoniae* was demonstrated [62]. Studies on the role of choline in *S. pneumoniae* have found the *lic* operon to be highly expressed in adherence, biofilm and brain infection [38], [57]. However, a direct link between matrix forming-opaque phenotype and *lic* operon was not explored. In this paper, we showed the important role for choline in biofilm formation and *in vivo* infection by *S. pneumoniae*.

Our data could also explain the role of the transparent and opaque variants in the disease process. We hypothesize that the transparent phenotype is recovered more frequently from the nasal surface than the opaque counterpart at least in part because it is unable to form a matrix. We also hypothesize that matrix production is not capsule-dependent because it has been shown that rough strains are still capable of forming a sticky matrix in a biofilm assay [6]. This hypothesis is further supported by our finding that total CPS produced by the WCH159 strains and their isogenic mutant derivatives were essentially similar. On the other hand, the sticky matrix produced by the opaque variant facilitates attachment to the mucosal surface, protects from attack by the immune system, and allows translocation to deeper host tissues. The preference for some strains to be in the transparent phase rather than opaque could be also the reason why some strains can invade the host while others cannot.

Recently, it was shown that early biofilm formation on microtiter plates does not correlate with the invasive disease potential of *S. pneumoniae* [63]. That study was performed using 18 h-old biofilms, where the bacteria had probably not produced an extracellular matrix. However, our study demonstrates that opaque pneumococci that produce extracellular matrix from a 4-day old biofilm culture are more capable of causing invasive disease. Thus, these differences could account for the discrepancy between the two studies. Our data also indicate that only the opaque phenotype is able to form extracellular matrix, and that the *lic* operon and *ciaRH* contribute to this process. We believe that the opaque variant is more capable of taking up choline from the environment due to the high expression of the *lic* operon, and this substance is then used in the formation of matrix.

These data have provided new insights into the molecular basis and significance of biofilm formation by *S. pneumoniae* and by pathogenic bacteria in general. This study has also increased our understanding of bacterial pathogenesis and opens a new vista to the development of alternative prophylactic and/or therapeutic strategies by targeting the extracellular matrix produced by pathogenic bacteria.

## Materials and Methods

### Ethics Statement

This study was conducted in compliance with the Australian code of practice for the care and use of animals for scientific purposes (7th Edition 2004) and the South Australian Animal Welfare Act 1985. All animal experiments were approved by the Animal Ethics Committee of the University of Adelaide (Project Number: S-86-2006).

### Bacterial strains and growth conditions

*S. pneumoniae*strains used in this study are listed in Table 3. Bacteria were sub-cultured from a frozen stock culture onto Todd-Hewitt agar supplemented with 0.5% yeast extract (THY agar) and 5,000 U of catalase, and incubated at 37°C in 95% air, 5% CO_2_ overnight. Colony morphology of variants on THY-catalase plates was determined under oblique transmitted illumination as previously described [14]. Pure cultures of the two variants selected on THY-catalase plates were subcultured three consecutive times, and remained stable upon subculturing. A single colony of each phenotype variant was then used to inoculate 5 ml of THY broth and incubated at 37°C in 95% air, 5% CO_2_. The cultures were grown until the mid-logarithmic phase (*A*_590_ = 0.2) in THY broth without catalase and used to inoculate the microtiter plates for biofilm formation assay. Before the assays, the growth rates of all strains were determined and these were essentially similar (not shown).

**Table 3:** *S. pneumoniae* strains used in this study.

Strain	Description	Sequence type (ST)^a^	Source/Reference
D39 (O/T)	Capsular serotype 2, opaque and transparent variants	595	[77]; This study
WCH16	Capsular serotype 6A clinical isolate	4966	Women's and Children's Hospital, North Adelaide, Australia
WCH43	Capsular serotype 4 clinical isolate	205	Women's and Children's Hospital, North Adelaide, Australia
WCH132	Capsular serotype 6B (L82016)	5400	D. E. Briles, UAB, Birmingham, AL, USA
WCH158	Capsular serotype 19F clinical isolate	1	Women's and Children's Hospital, North Adelaide, Australia
WCH159 (O/T)	Capsular serotype 19F clinical isolate, opaque and transparent variants	1	Women's and Children's Hospital, North Adelaide, Australia; This study
WCH159 Δ*ciaRH* (O/T)	CiaRH in-frame deletion replacement mutant (Ery^R^), opaque and transparent variants	1	This study
WCH159 Δ*licD*2 (O/T)	LicD2 in-frame deletion replacement mutant (Spec^R^), opaque and transparent variants	1	This study
D39 Δ*ciaRH* (O/T)	CiaRH in-frame deletion replacement mutant (Ery^R^), opaque and transparent variants	595	This study
D39 Δ*licD*2 (O/T)	LicD2 in-frame deletion replacement mutant (Spec^R^), opaque and transparent variants	595	This study

a The sequence types of the strains was characterized as described in the Multi Locus Sequence Typing (MLST) database.

### Biofilm assays

Static biofilm assay was performed by growing bacteria in 96-well flat-bottom polystyrene plates (Sarstedt). Approximately 1×10^5^ mid-exponential phase pneumococci were inoculated 1∶10 into 1∶3 diluted THY medium. The plates were incubated at 37°C in a CO_2_-enriched atmosphere for 1, 2, 3, 4 or 5 days. To permit the extension of the experiment for days, medium was changed daily. To collect bacteria attached to the plastic substratum, plates were washed 3 times and filled with 100 µl of fresh THY medium, sealed and floated on a sonicating water bath (Transonic 460/H ultrasonic bath) and sonicated for 3 sec at 35 KHz. To evaluate and quantify the colony morphology variants, planktonic and sessile cultures were harvested at various time points, serially diluted, and plated onto THY-catalase plates. At each time point, bacterial morphology and formation of aggregates was checked by light microscopy. As already reported [33], the time of sonication was enough to disperse bacterial aggregates. At 4 days post incubation, biofilm was used for mouse challenge experiments, RNA extraction, and adherence assays. Bacteria were harvested from the planktonic phase and then from the bottom of microtiter plates by sonication. All types of inocula were resuspended in THY, stored as frozen aliquots, and diluted in THY for challenge and adherence assays. For RNA extraction, samples were immediately processed after collection.

### Scanning electron microscopy (SEM)

SEM was performed on 4 day biofilms formed on plastic coverslips (0.2 mm thick, 13 mm diameter; Nunc) in 24-well microtiter plates, seeded with 1 ml of cell suspension, as above. Plates were statically incubated at 37°C for 4 days and media changed daily, as described above. At the end of the incubation, the coverslips were washed with phosphate-buffered saline (PBS), and fixed O/N at 4°C with a solution of 3% gluteraldehyde (Sigma). The coverslips were washed three times with PBS, 4% sucrose, and post-fixed with 0.1% osmium tetraoxide for 1 h. After 3 washes in the same buffer, a series of dehydration steps was performed using graded ethanol baths (70%, 90% and 100%, three times for 10 min each). After critical point drying and coating by gold sputter, samples were examined with a Philips XL30 FEGSEM scanning electron microscope. For each experiment, 3 replicates resulting from 3 independent inocula were analyzed.

### Adherence assays

A549 human lung alveolar carcinoma (type II pneumocyte) and nasopharyngeal human carcinoma epithelial (Detroit 562) cell lines were used for adherence assays. Cells were grown in Dulbecco's modified Eagle's medium (DMEM) and Minimal essential medium with Earle's salt (EMEM), respectively, and supplemented with 10% fetal calf serum (FCS) and maintained in the appropriate medium with 1% FCS. Cells were grown in 75-cm^2^ tissue culture bottles (Becton Dickinson) at 37°C in a 5% CO_2_ atmosphere. Confluent cells were seeded into 6-well tissue culture trays (Becton Dickinson) for use in the adherence assays. Opaque and transparent variants of strain WCH159 were grown in a static biofilm assay and used for adherence assays. Cultures were diluted to the appropriate density (5×10^6^ CFU/ml) in DMEM or EMEM plus 10% FCS (without antibiotics) and 1 ml aliquots were inoculated onto washed A549 or Detroit 562 cells. After incubation for 2 h at 37°C, the wells were washed three times with PBS and cells were detached from the plate by treatment with 100 µl of 0.25% trypsin-0.02% EDTA. Appropriate dilutions of the cultures were plated on blood agar to determine the number of adherent bacteria. Assays were performed in quadruplicate from four independent experiments. For scanning electron microscopy, tissue cultures infected with the same bacteria were grown on coverslips and treated as described above.

### Intranasal challenge of mice

Groups of either 15 or 30 female CD-1 mice (5–6 weeks old) were anesthetized by intraperitoneal injection of sodium pentobarbitone (Nembutal; Rhone-Merieux) at a dose of 66 µg per g of body weight and challenged intranasally with 50 µl of bacterial suspension containing approximately 5×10^6^ CFU in THY medium. The challenge dose was confirmed by plating serial dilutions of the inocula on blood agar. At 24, 48 and 96 h post challenge, 5 or 10 mice infected with each strain were euthanased by CO_2_ asphyxiation, and nasal wash, nasal tissue, lung, blood and brain samples were plated on blood agar to enumerate pneumococci present in each niche and on THY+catalase plates to check colony morphology, as described previously [64]. The Ethics Committee of The University of Adelaide approved all animal experiments.

### RNA extraction and microarray analysis

RNA was isolated from biofilm samples by using acid-phenol-chloroform-isoamyl alcohol (125∶24∶1 pH 4.5; Ambion) and treated as described previously [64], [65]. Microarray experiments were performed on whole genome *S. pneumoniae* PCR arrays based on TIGR4 and R6 annotations [66]. Array slides were obtained from the Bacterial Microarray Group at St George's, University of London. The array design is available in BµG@Sbase (Accession No. A-BUGS-14; http://bugs.sgul.ac.uk/A-BUGS-14) and also ArrayExpress (Accession No. A-BUGS-14).

Fluorescently labelled cDNAs were hybridized to the surface of the microarray as described previously [67], [68]. The Spot plugin (CSIRO, Australia) within the R statistical software package (http://www.R-project.org) and the Limma plugin for R [69] were used for data processing and statistical analysis. Ratio values were normalized using the print-tip Loess normalization routine [70] and a linear model fitted to determine a final expression value for each mRNA [71]. These statistics were used to rank the mRNAs from those most likely to be differentially expressed to the least likely using false discovery rate of *P*<0.05. A fold change of ≥2.0 in gene expression with statistical significance (*P*≤0.01) was classified as being significantly changed. In this study, nine independent hybridizations, including one labelled by dye reversal, using RNA samples isolated from four separate assays were performed for each incubation time point.

### Real-time RT-PCR

For a subset of selected genes, differences in levels of expression obtained by microarray analysis were validated by one-step relative quantitative real time RT-PCR in a Roche LC480 Real-Time Cycler, essentially as described previously [67]. The specific primers used for the various RT-PCR assays are listed in Table S2 and were used at a final concentration of 200 nM per reaction. As an internal control, primers specific for the 16S rRNA were employed. Amplification data were analyzed using the comparative critical threshold (2^-ΔΔCT^) method [72].

### Mutant construction and transformation

Mutants were constructed by gene SOEing essentially as described previously [73], [74]. Deletion of *licD*2 and the *ciaRH* operon was achieved by replacing the respective genes in WCH159, in-frame, with a spectinomycin- or erythromycin-resistance cassette, respectively. The primers used are listed in Table 4. Opaque and transparent bacteria were transformed using competence stimulating peptide (CSP)-1 or 2, essentially as described previously [74]. Mutants were selected by multilayer plating as previously described and verified by sequencing using primers flanking the gene in question [74].

**Table 4:** Primers used in this study.

Primer^a^	Sequence (5′→3′)
*ciaRH* Flank F	ATGCACATGCTTCGCCGTTGGC
*ciaRH* Ery F	TTGTTCATGTAATCACTCCTTCTATTAAAACTATTATACCAAATTTGCC
*ciaRH* Ery R	CGGGAGGAAATAATTCTATGAGTCCAACTGGGGCGATATTTTGGAT
*ciaRH* Flank R	GGGCCATTTGGCACCTGCAAAC
*licD*2 Flank F	CTCGTCTCAGTTACTATCTGGGGA
*licD*2 Spec F	TATGTATTCATATATATCCTCCTCTTTATAAGCGTGAAATTCATGACTGTAG
*licD*2 Spec R	AAATAACAGATTGAAGAAGGTATAATTGAGGGGGATTATACAGACTAC
*licD*2 Flank R	GACCATCCTTGGTACACAGGTTGC
JM214	GAGGAGTGATTACATGAAACAA
JM215	CTCATAGAATTATTTCCTCCC
JM253	GAGGAGGATATATATGAATACATACG
JM254	TTATACCTTCTTCAATCTGTTATTTAAATAGTTTATAGTTA

a Primers for *ciaRH* and *licD*2 were derived from the *S. pneumoniae* G54 (serotype 19F) genome as deposited in the Kyoto Encyclopedia of Genes and Genomes (KEGG) database.

### Multi-locus sequence typing (MLST)

For MLST analysis, the *aroE*, *gdh*, *gki*, *recP*, *spi*, *xpt*, and *ddl* genes for the *S. pneumoniae* strains were PCR-amplified and sequenced. The sequence type (ST) of strains was obtained from the MLST database (http://www.mlst.net) based on the resulting allelic profiles.

### Western blotting

All cultures of transparent and opaque WCH159 grown in the 4-day static biofilm model were analyzed for the presence of TA by Western blotting with TEPC-15 (a mouse anti-P-cho monoclonal antibody). Prior to SDS-PAGE and immunoblotting, samples were placed in 2 ml vials containing 2.8 mm ceramic beads and pulsed in a Precellys 24 tissue homogeniser (Bertin Technologies) at 3 cycles of 30 seconds at 5000 rpm to disperse any bacteria that might be embedded in the matrix. Samples (containing approx 5×10^6^ CFU/ml bacteria) were then centrifuged at 18,000× *g* for 3 min to pellet the bacteria, and aliqouts of the supernatant fractions of sessile opaque samples subjected to proteinase K treatment. The supernatant and pellet fractions were then resuspended in sample loading buffer, electrophoresed, transferred unto nitrocellulose and blotted with TEPC-15. The secondary antibody was goat-anti mouse IgA conjugated to alkaline phosphatase (Zymed laboratories, San Francisco, CA, USA).

### ELISA for CPS quantitation

Total CPS produced by WCH159 strains and their isogenic mutant derivatives was quantified by ELISA using a modification of the method described previously [75]. Briefly, serial two-fold dilutions of either purified type 19F CPS (American Type Culture Collection, USA) standard (starting concentration of 10 µg/ml) or total CPS preparations of bacteria [76] were coated unto poly-L-lysine treated Nunc MaxiSorp® flat-bottom 96 well plates overnight at 4°C. After blocking with 1% foetal calf serum, the samples were reacted with a 1∶10,000 dilution of group 19 typing sera (Statens Seruminstitut, Copenhagen, Denmark) for 4 h. The plates were washed five times in wash buffer, and then reacted with a 1∶20,000 dilution of goat anti–rabbit IgG alkaline phosphatase conjugate overnight at 4°C. After extensive washing, the plates were developed using alkaline phosphatase substrate (Sigma) in diethanolamine buffer, and read at *A*_405 nm_.

### Statistical analyses

Unless where otherwise stated, statistical analyses for *in vitro* and *in vivo* experiments were carried out using Student's *t*-test, two-tailed (GraphPad Prism version 5). A *P* value of <0.05 was considered significant.

## Supporting Information

Figure S1 Colony morphology analysis of pneumococci on THY-catalase plates after 4 day static biofilm assay, showing: (**A**) a high percentage of transparent variants (90%) in the low biofilm-forming strains (WCH16, WCH43 and WCH132), and (**B**) a high percentage (approx. 90%) of opaque variants (WCH158, WCH159 and D39) at day 7.
(PDF)

Figure S2 Static biofilm assay of opaque and transparent pneumococci. Opaque variants of D39, WCH43, WCH159, WCH158, WCH132 and WCH16 were able to form stable biofilms over the incubation period, peaking at day 4 (**A**), whereas the transparent variants of D39, WCH16 and WCH43 were impaired in their abilities to form biofilms (**B**).
(PDF)

Figure S3 Western blotting of supernatant and pellet fractions of samples from a 4 days static biofilm cultures transparent and opaque WCH159 with TEPC-15 (a mouse anti-phosphorylcholine monoclonal antibody). Lanes: 1, liquid opaque bacteria; 2, liquid transparent bacteria; 3, pellet fraction of sessile opaque bacterial culture; 4, supernatant fraction of sessile opaque bacterial culture; 5, pellet fraction of sessile opaque bacterial culture; 6, supernatant fraction of sessile opaque bacterial culture; 7, supernatant fraction of sessile opaque bacterial culture, proteinase K-treated for 1 h.
(PDF)

Table S1 RT-PCR comparisons of gene expression of *S. pneumoniae* WCH159 transparent (T)/opaque (O) and sessile (S)/planktonic (P) variants.
(DOC)

Table S2 Oligonucleotide primers.
(DOC)
