# Supplementary figures and images for: Extracellular Matrix Formation Enhances the Ability of Streptococcus pneumoniae to Cause Invasive Disease
Source: PLoS One. 2011 May 18;6(5):e19844. doi: 10.1371/journal.pone.0019844 (PMC3097209; doi:10.1371/journal.pone.0019844)

**A**

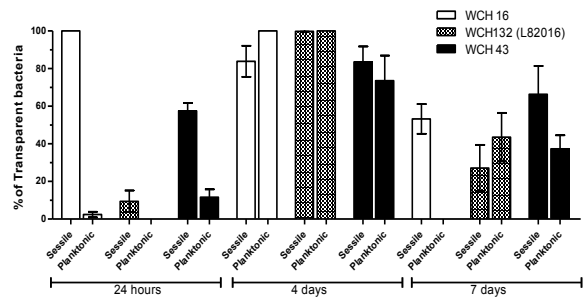

**B**

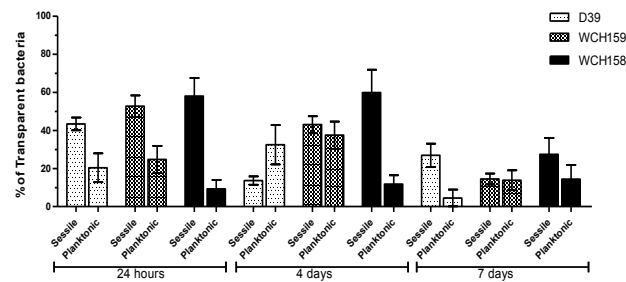

Supplement: Figure S1 — Colony morphology analysis of pneumococci on THY-catalase plates after 4 day static biofilm assay, showing: (A) a high percentage of transparent variants (90%) in the low biofilm-forming strains (WCH16, WCH43 and WCH132), and (B) a high percentage (approx. 90%) of opaque variants (WCH158, WCH159 and D39) at day 7. (PDF) [file pone.0019844.s001.pdf]

**A****Biofilm formation from Opaque cultures**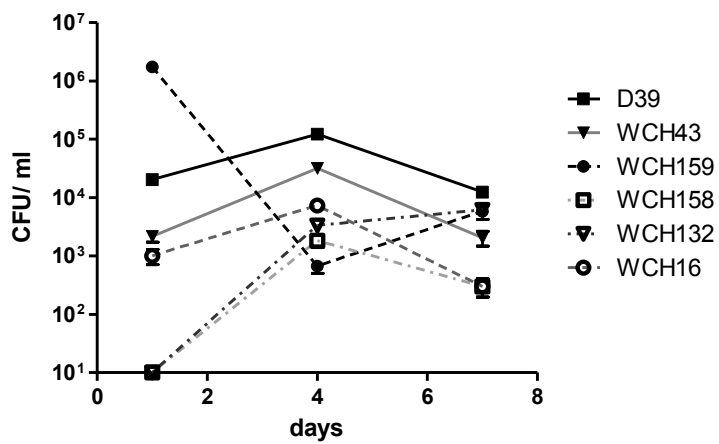**B****Biofilm formation from Transparent cultures**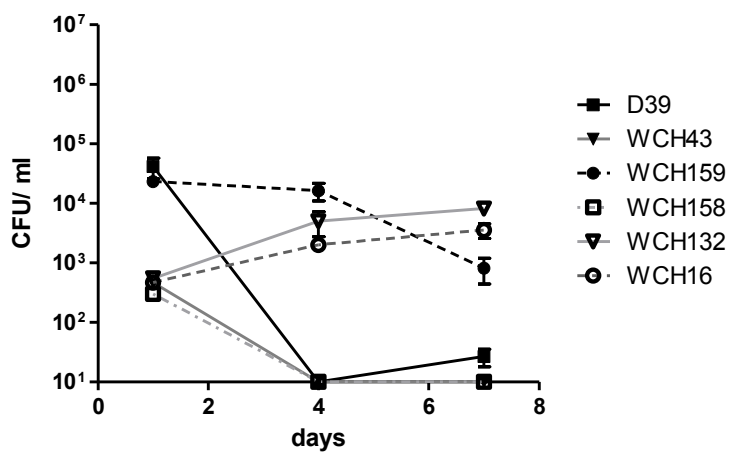

Supplement: Figure S2 — Static biofilm assay of opaque and transparent pneumococci. Opaque variants of D39, WCH43, WCH159, WCH158, WCH132 and WCH16 were able to form stable biofilms over the incubation period, peaking at day 4 (A), whereas the transparent variants of D39, WCH16 and WCH43 were impaired in their abilities to form biofilms (B). (PDF) [file pone.0019844.s002.pdf]

1 2 3 4 5 6 7

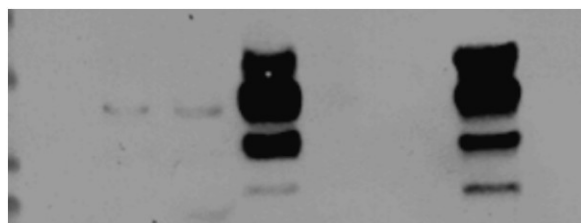

Supplement: Figure S3 — Western blotting of supernatant and pellet fractions of samples from a 4 days static biofilm cultures transparent and opaque WCH159 with TEPC-15 (a mouse anti-phosphorylcholine monoclonal antibody). Lanes: 1, liquid opaque bacteria; 2, liquid transparent bacteria; 3, pellet fraction of sessile opaque bacterial culture; 4, supernatant fraction of sessile opaque bacterial culture; 5, pellet fraction of sessile opaque bacterial culture; 6, supernatant fraction of sessile opaque bacterial culture; 7, supernatant fraction of sessile opaque bacterial culture, proteinase K-treated for 1 h. (PDF) [file pone.0019844.s003.pdf]
