# Supplementary material for: Extracellular Matrix Formation Enhances the Ability of Streptococcus pneumoniae to Cause Invasive Disease
Source: PLoS One. 2011 May 18;6(5):e19844. doi: 10.1371/journal.pone.0019844 (PMC3097209; doi:10.1371/journal.pone.0019844)
Supplement: Table S1 — RT-PCR comparisons of gene expression of S. pneumoniae WCH159 transparent (T)/opaque (O) and sessile (S)/planktonic (P) variants. (DOC) [file pone.0019844.s004.doc]

**Table S1.** RT-PCR comparisons of gene expression of *S. pneumoniae* WCH159 transparent (T)/opaque (O) and sessile (S)/planktonic (P) variants.

| Gene ID  (TIGR4)^a^ | Gene ID  (G54)^b^ | Gene annotation | FOLD CHANGE | | | |  |
| --- | --- | --- | --- | --- | --- | --- | --- |
|  |  |  | sO/ST | PO/PT | SO/PO | st/PT | Tle/OLe^c^ |
| Sp_1937 | SpG_1847 | Autolysin (LytA) | -6.72 | -1.35 | 1.09 | 0.22 |  |
| Sp_2190 | SPG_2135 | Choline binding protein A | -6.92 | -1.56 | -1.34 | 0.30 |  |
| Sp_0117 | SPG_0121 | Pneumococcal surface protein A | -10.32 | -1.66 | -15.31 | -7.26 |  |
| Sp_2136 | SPG_ [2074](http://www.genome.jp/dbget-bin/www_bget?spx:SPG_2074) | Choline binding protein PcpA | -5.26 | -1.98 | 0.35 | 0.13 |  |
| Sp_1272 | SPG_ [1166](http://www.genome.jp/dbget-bin/www_bget?spx:SPG_2074) | Polysaccharide biosynthesis protein (TacF) | 7.16 | 2.13 | -0.85 | -1.03 |  |
| Sp_1269 | SPG_1163 | Choline kinase (LicA) | 5.87 | 1.75 | -0.82 | 0.24 |  |
| Sp_1274 | SPG_1168 | LicD2 protein |  |  |  |  | 2.00 |
| Sp_0798 | SPG_0728 | Two component System 05 Response Regulator |  |  |  |  | 2.36 |
| Sp_2239 | SPG_2188 | Serine protease HtrA |  |  |  |  | 2.17 |

^a, b^ Gene IDs were obtained from the *S. pneumoniae* TIGR4 (serotype 4) and G54 (serotype 19F) genomes as deposited in the Kyoto Encyclopedia of Genes and Genomes (KEGG) database.

^c^  TLE = Transparent late exponential phase culture; OLE = Opaque late exponential phase culture.
