# Supplementary material for: Extracellular Matrix Formation Enhances the Ability of Streptococcus pneumoniae to Cause Invasive Disease
Source: PLoS One. 2011 May 18;6(5):e19844. doi: 10.1371/journal.pone.0019844 (PMC3097209; doi:10.1371/journal.pone.0019844)
Supplement: Table S2 — Oligonucleotide primers. (DOC) [file pone.0019844.s005.doc]

**Table S2.** Oligonucleotide primers.

| Primer^a^ | Sequence | Annotation |
| --- | --- | --- |
| MalX For | ACTGCTGATAAGCCTGCTGA | SPG_2045 |
| MalX Rev | CCTCCTAGAGCATCACCAGT |  |
| MalT For | TGCAAGTAGTCACGAGCATCAA | SPG_1505 |
| MalT Rev | TATCCGTCCAGAAGACGTGA |  |
| Pts For | GCATTATGGTTGTAGACGAC | SPG_0065 |
| Pts Rev | AACCAAGGAAGCGGTCTGGT |  |
| Omp For | AGGCAGGTTATCAGGTCTTG | SPG_0084 |
| Omp Rev | AATCTTGTCCTGTTCACTGG |  |
| ArcA For | TACTTCACGGCATCAAGG | SPG _2088 |
| ArcA Rev | ATGGATACTCTGATTCAACT |  |
| LicD2 For | ACCATGCTTGGAGCCATCCG | SPG_1168 |
| LicD2 Rev | ATGGAAGTACCAAGAAGAT |  |
| CiaR For | gatggagaagaaggtc | SPG_0728 |
| CiaR Rev | gtcataatcagaactgg |  |
| HtrA For | GTTTCGCAATTCCTGCAAAT | SPG_2188 |
| HtrA Rev  LytA For  LytA Rev  CbpA For  CbpA Rev  PspA For  PspA Rev  PcpA For  PcpA Rev  LicA For  LicA Rev  TacF For  TacF Rev | TGGTGTAGTTGTTCGTTCGG  ctatgcagcggttgaactga  ccacatggtctgagtggttg  agacttccgtccagctttca  cattgccagttcatccattg  ggcagaagaagctgagttgg  agcttgctcaccgtttgagt  tgaatcgtttgcctcagttg  gtttaatcgcatccgcaaat  cgatttggtgcctgaaaact  accggtgtttggtcactctc  atgggtcaatgaagctctgg  atggctgtcagagggagaaa | SpG_1847  SPG_2135  SPG_0121  SPG_[2074](http://www.genome.jp/dbget-bin/www_bget?spx:SPG_2074)  SPG_[1166](http://www.genome.jp/dbget-bin/www_bget?spx:SPG_2074)  SPG_1163 |

^a^ Primers for RT-PCR validation were derived from the *S. pneumoniae* G54 (serotype 19F) genome as deposited in the Kyoto Encyclopedia of Genes and Genomes (KEGG) database.
